# Diel investments in metabolite production and consumption in a model microbial system

**DOI:** 10.1038/s41396-021-01172-w

**Published:** 2021-12-17

**Authors:** Mario Uchimiya, William Schroer, Malin Olofsson, Arthur S. Edison, Mary Ann Moran

**Affiliations:** 1 Department of Marine Sciences, University of Georgia, Athens, GA 30602 US; 2 Complex Carbohydrate Research Center, University of Georgia, Athens, GA 30602 US; 3 Swedish University of Agricultural Sciences, Department of Aquatic Sciences and Assessment, Uppsala, Sweden

**Keywords:** Biogeochemistry, Metabolomics

## Abstract

Organic carbon transfer between surface ocean photosynthetic and heterotrophic microbes is a central but poorly understood process in the global carbon cycle. In a model community in which diatom extracellular release of organic molecules sustained growth of a co-cultured bacterium, we determined quantitative changes in the diatom endometabolome and the bacterial uptake transcriptome over two diel cycles. Of the nuclear magnetic resonance (NMR) peaks in the diatom endometabolites, 38% had diel patterns with noon or mid-afternoon maxima; the remaining either increased (36%) or decreased (26%) through time. Of the genes in the bacterial uptake transcriptome, 94% had a diel pattern with a noon maximum; the remaining decreased over time (6%). Eight diatom endometabolites identified with high confidence were matched to the bacterial genes mediating their utilization. Modeling of these coupled inventories with only diffusion-based phytoplankton extracellular release could not reproduce all the patterns. Addition of active release mechanisms for physiological balance and bacterial recognition significantly improved model performance. Estimates of phytoplankton extracellular release range from only a few percent to nearly half of annual net primary production. Improved understanding of the factors that influence metabolite release and consumption by surface ocean microbes will better constrain this globally significant carbon flux.

## Introduction

The transfer of organic carbon from phytoplankton to bacteria via a pool of labile dissolved compounds is a key process in global carbon cycling, involving up to a third of fixed carbon [1, 2]. One important but poorly quantified mechanism for this transfer is the extracellular release of organic compounds by living phytoplankton. Indeed, estimates of the proportion of net primary production (NPP) released extracellularly range from 4 to 47% [3], and of the proportion of heterotrophic bacterial carbon demand supported by extracellular release from 2 to 50% [1]. In part, this largely unconstrained flux can be traced to the difficulties inherent in characterizing labile organic molecules dissolved in surface seawater, such as short turnover times due to rapid bacterial uptake and low concentrations due to efficient scavenging (nmol L^−1^ to pmol L^−1^; refs. [4, 5]). Moreover, organic molecules taken up by bacteria become rapidly untraceable because of transformation and respiration inside bacterial cells [2]. Although the dissolved organic carbon (DOC) link between marine phytoplankton and bacteria has long been of interest [6–8], identifying the specific metabolites responsible and measuring their flux is indeed challenging.

Phytoplankton metabolite synthesis exhibits diel cycles in the surface ocean, coordinated with the availability of light energy [9–11]. Similar diel cycles of activity in heterotrophic bacterial communities have also been observed [12–14], often lagging peak phytoplankton activity by a few hours [14, 15]. Gene expression data are providing detailed views of this diel synchronicity between phytoplankton and bacteria in oligotrophic and coastal marine communities. For example, phytoplankton carbon fixation and photosystem gene expression coordinates with bacterial substrate uptake (amino acid and sugar transporters) and citric acid cycle gene expression [16, 17]. Extracellular release of phytoplankton metabolites is the process most likely to underlie these matched patterns.

Intracellular phytoplankton metabolite pools (endometabolites) are the presumptive substrates for the heterotrophic bacteria supported by extracellular release. Yet how faithfully phytoplankton internal concentrations predict exometabolite availability depends on the mechanism of release [3, 18]. In the simplest mechanism, differences in metabolite concentration between phytoplankton cells and ambient seawater drive diffusion [19] (i.e., passive diffusion mechanism), in which case metabolite release to heterotrophic bacteria is largely controlled by intracellular metabolite concentrations of the phytoplankton. Alternatively, active excretion of metabolites to maintain cellular balance can occur by overflow pathways [20], for example to manage redox state [11] or photorespiration (i.e., physiological balance mechanism). Finally, metabolites may be synthesized and excreted in response to associated microbes, potentially to sustain mutualisms or mount defenses [21–23] (i.e., interaction response mechanism).

Here we determined the correspondence between phytoplankton intracellular pools and heterotrophic bacterial substrate availability by examining diel patterns of endometabolites and transcripts, respectively. A model community was established in which marine diatom *Thalassiosira pseudonana* CCMP1335 [24] was the only source of substrates to bacterium *Ruegeria pomeroyi* DSS-3 [25]. As diatoms contribute up to 40% of primary production in the surface ocean [26], and *R. pomeroyi* belongs to a taxon that often dominates diatom bloom communities [27, 28] and responds sensitively to external resources [29–31], this simple community represents a relevant phytoplankton-bacteria association in the surface ocean. Over two day-night cycles, we contemporaneously assayed phytoplankton endometabolite pools by NMR spectroscopy and bacterial metabolite consumption using transcript proxies, and assessed links between the two. Transcript abundance was quantified as the number of mRNA molecules per bacterial cell, enabled by the use of internal mRNA standards; this approach yielded the number of transcripts harbored by a cell for a given gene, matching absolute quantitation in the metabolite data and eliminating ambiguities inherent in proportional expression data [32–34]. The quantitative chemical-biological analytical framework applied to this model system enabled us to assess proposed mechanisms underlying temporal links between microbial autotrophs and heterotrophs via extracellular release of labile metabolites.

## Materials and methods

### Diel experiment

An axenic strain of marine diatom *Thalassiosira pseudonana* CCMP1335 was cultured at 18 ^o^C in three replicate 15-L polycarbonate bottles containing 10 L of L1 medium [35] in which NaH^13^CO_3_ (Cambridge Isotope Laboratories, CLM-441) was the source of inorganic carbon (labeling efficiency, 78% of C atoms). The light cycle consisted of 16 h light, during which light intensity varied gradually between 0 and 150 µmol photon m^−2^ s^−1^ with a maximum intensity at noon, followed by 8 h of dark. Axenic *T. pseudonana* cultures were pre-incubated for 6 days to achieve cell numbers required for metabolite analysis. The cultures were inoculated with bacterial strain *Ruegeria pomeroyi* DSS-3 that was grown overnight at 30^o^C on ½ YTSS liquid medium and washed three times in L1 medium (final concentration, 10^6^ bacterial cells mL^−1^). Co-cultures (*n* = 3) were pre-incubated for two days, after which samples were collected every 6 h over the next 48 h for bacterial mRNA sequencing, phytoplankton and bacterial cell counts, and phytoplankton endometabolome analysis.

### Bacterial effect experiment

*T. pseudonana* CCMP1335 and *R. pomeroyi* DSS-3 co-cultures were prepared as in the diel experiment except that additional treatment was established in which the diatom remained axenic (*n* = 3 for each). Diatom cells were collected at the 48 h sample time and metabolite concentrations were compared between treatments. Incubation periods, culture media, sample processing protocols, and metabolite analysis schemes were the same as those used in the main experiment.

### Light effects experiment

One-week stationary-phase axenic *T. pseudonana* CCMP1335 grown under similar conditions were sequentially filtered through GF/F filters (Whatman, Maidstone, UK) and 0.2-µm-pore-size PCTE membrane filters (Poretics, Swedesboro, NJ) to remove cells. *R. pomeroyi* DSS-3 cells prepared as described above were added to the diatom-free filtrate and incubated under light intensities of 150 (100% treatment), 75 (50% treatment), or 0 µmol photon m^−2^ s^−1^ (0% treatment), corresponding to light levels at noon, mid-morning and mid-afternoon, and night in the diel experiment (*n* = 3 for each). Temperature monitoring indicated only a minor temperature increase of 0.5^o^C in the 100% treatment relative to 50% and 0% treatments. After 4 h, samples were collected for bacterial RNA analysis and cell counts.

### Diatom endometabolome analysis

Diatom cells were collected by filtering 500 mL of culture onto 2.0-µm-pore-size PCTE membrane filters (Isopore; MilliporeSigma, Burlington, MA) using <10 inHg pressure and stored at −80^o^C until processing. Cells were removed from filters by sonication in ultra-pure water (MilliporeSigma) and concentrated by freeze-drying [36]. Pellets were mixed with 600 µL of sodium phosphate butter (pH 7.4) with an internal standard of 2,2-dimethyl-2-silapentane-5-sulfonate-d_6_ (DSS) (1 mmol L^−1^), vortexed for 5 min, and centrifuged at 20,800 *rfc* for 10 min, after which supernatants were transferred to 5-mm NMR tubes (Bruker, Billerica, MA). Processing was conducted at 4^o^C. A blank sample was also included for quality control. Metabolites were analyzed by NMR spectroscopy using a Bruker AVANCE III 800 MHz 5 mm TCI cryoprobe, 800 MHz 1.7 mm TCI cryoprobe, and 600 MHz 5 mm TXI probe. Pulse programs of ^1^H-^13^C heteronuclear single quantum correlation (HSQC; Bruker program hsqcetgpprsisp2.2), ^1^H-^13^C HSQC-total correlation spectroscopy (HSQC-TOCSY; hsqcdietgpsisp.2), and ^1^H-^13^C heteronuclear multiple bond correlation (HMBC; hmbcetgpl2nd) were used. Data were deposited to Metabolomics Workbench (Project ID, PR001019). Data processing was carried out with TopSpin 4.0.3 (Bruker), and peak intensity was extracted using rNMR 1.1.9 [37]. Metabolites were annotated based on chemical shift (HSQC) and coupling information (HSQC-TOCSY and HMBC). The Human Metabolome Database (HMDB) [38] and the Biological Magnetic Resonance Bank (BMRB) [39] were used as reference databases, and additionally the Carbohydrate Structure Database (CSDB) [40] for polysaccharides. Three compounds of interest that are not in these databases were annotated either by obtaining original spectra from chemical standards (DHPS and DMSP; ref. [41]) or based on literature values (homarine; ref. [42]). A confidence level of annotation was assigned to each metabolite [43] (Table S1) where 1 = putative compounds with functional group information; 2 = partially matched to HSQC chemical shift information in the databases or literature; 3 = matched to HSQC chemical shift; 4 = matched to HSQC chemical shift and validated by HSQC-TOCSY or HMBC; 5 = validated by original spectra from chemical standards. Detailed parameter settings are presented in Table S2, with additional information in Metabolomics Workbench. Spectra were standardized to DSS, peak intensities were normalized to cell counts, and data were auto-scaled and presented as *Z*-scores. Since we did not conduct additional spiking experiments to quantify compounds, we only focus on the difference of the values between samples, and not between compounds. Temporal variations in metabolites were analyzed by extracting peaks using variance-sensitive clustering [44]. The optimal cluster number was selected based on minimum centroid distance and the Xie-Beni index values for the dataset [44]. Background signals originating from labware and solvent were also corrected. Background signals originating from bacteria trapped on the 2.0-µm filters were extremely low in the diatom endometabolite fraction (Supplementary Methods). Periodicity of the temporal patterns for compounds was analyzed using a rhythmicity analysis package RAIN (1.18.0; ref. [45]) in R software (version 3.6.1). Heatmaps were created using the CirHeatmap function (version 1.7) in MATLAB (Mathworks, Natick, MA) [46].

### mRNA analysis

For the diel experiment, samples were sequentially filtered through 2.0-µm pore-size PCTE filters (Isopore; MilliporeSigma) to remove diatom cells and 0.2-µm pore-size PES filters (Supor; Pall, New York) to retain bacterial cells. RNA was extracted from the bacterial filters with a phenol-chloroform-isoamyl method [47], which has a better RNA recovery for co-culture samples. Two internal mRNA standards (size, 1,000 nt) were added to each sample before extraction for determining their recovery in the sequence data [48]. For the direct light experiment, samples were filtered through the 0.2-µm pore-size filters to retain bacterial cells. RNA was extracted using the RNeasy Mini Kit (QIAGEN, Hilden, Germany) after cutting filters into pieces under sterile conditions and shaking with 0.5 mL of 0.1-mm zirconia/silica beads (BioSpec Products, Bartlesville, OK) in 1 mL of Denaturation/Lysis Solution (Life Technologies, Carlsbad, CA) for 15 min. For both sample types, filtration was completed within 15 min of collection, filters were flash frozen in liquid nitrogen and then stored at −80^o^C, DNA was removed (Turbo DNA-free Kit, Ambion, Austin, TX), rRNA was depleted (Ribo-Zero rRNA Removal Kit; Illumina, San Diego, CA), and mRNA was purified (RNA Clean & Concentrator-5; Zymo Research, Irvine, CA). The number of bacteria extracted was ~10^9^ cells.

Sequencing using NextSeq 550 (Illumina) SE50 averaged 19.2 x 10^6^ reads per sample (Table S3). rRNA reads were identified by blast+ (NCBI 2.7.1 and 2.8.1 for the direct light experiment and the diel experiment, respectively) against an rRNA sequence database and removed; rRNA contamination averaged 17.5% of reads. Recovery of the two internal standards was highly consistent (Pearson’s *r* = 0.96; *p* < 0.001; *n* = 26), accounting for 2.2% of mRNA reads per library (Table S3). Remaining reads were mapped to the *R. pomeroyi* genome and quantified using HTSeq [49]. Differentially expressed genes were identified in pairwise comparisons of sampling times (diel experiment) or light levels (direct light experiment) using MATLAB for absolute analysis and DESeq2 [50] for relative analysis. One of the replicate samples from the initial time point of the experiment was lost; otherwise, *n* = 3 for all analyses. All other statistical analyses were conducted using MATLAB. Fold-change values and temporal pattern categories for all genes are reported in Table S4.

### Cell counts

A 0.5 mL aliquot of culture fixed with glutaraldehyde (final concentration, 1%) was kept at −80^o^C until analysis. Samples were thawed, stained with SYBR Green I (Thermo Fisher Scientific, Waltham, MA; final concentration, 5 x 10^−4^ dilution of commercial stock), and injected into a CytoFLEX flow cytometer (Beckman Coulter, Brea, CA). For phytoplankton counts, samples were analyzed without staining. Data were analyzed using CytExpert (Beckman Coulter) and cell density was calculated based on a separate run of a known concentration of bead standards (Beckman Coulter).

### Model development

A model was written in R version 3.6.1 with three state variables representing the phytoplankton endometabolome (*P*), the medium exometabolome (*E*), and the bacterial endometabolome pools (*B*). The time evolution of these pools was calculated at 0.1 h intervals. The base model was written to represent only diffusive and fundamental physiological processes, which is largely a passive model, given in the following differential equations:

$$\delta _tP = N - T - R$$

$$\delta _tE = R - U$$

$$\delta _tB = U - C$$

*N* is the metabolite biosynthesis rate, derived from light intensity expressed as a cosine function. *T* is rate at which endometabolites are allocated for biomass and energy generation by phytoplankton cells, calculated as a constant fraction of *P* from the previous interval. *R* is release rate of endometabolites from the phytoplankton cell by simple diffusion. *U* represents bacterial uptake rate from the exometabolome following Michaelis-Menten kinetics. C represents catabolism rate of the metabolite within the bacterial endometabolome, with a constant fraction lost each interval. See Supplemental Methods for information on how variables *N*, *R*, *T*, *U*, and *C* were derived.

In addition to the base model, three active terms could be added to the model to represent processes beyond those of simple diffusion and fundamental physiology. The first active term was fixation-irradiance oscillation, *o*, which represents the process of asymmetric carbon fixation in response to increasing versus decreasing light intensity and impacts the calculation of metabolite production, *N*. During periods of increasing irradiance (pre-noon), carbon fixation increases proportionally to irradiance, but during periods of decreasing irradiance (post noon), carbon fixation declines more rapidly than irradiance (see Supplemental Methods for details). The second active term was homeostasis, *h*, representing the increased phytoplankton excretion of metabolite, *R*, to maintain physiological homeostasis during periods of high light intensity. When *h* is incorporated into the model, *R* becomes a function of the rate of metabolite production, *N*, following the irradiance curve, such that *R* increases as *N* increases. The final active term was bacterial response, *b*, representing a recognition response in the phytoplankton to the presence of bacteria. The *b* term is multiplicative factor affecting the value of *N* and representing a change in metabolite production in response to the presence of a co-cultured bacterium.

The model was run for a simulated length of 10 d to replicate experimental conditions. During the first 6 d of ‘axenic growth’ the values *B* and *U* were set to zero, followed by ‘inoculation’ with addition of *B* and *U* functions for the final 4 d of the modeled experiment. Values for *P* and *U* from the final 2 d of model output were used to compare to experimentally measured endometabolome and transcriptome data, respectively.

The model was fit to each matched metabolite/gene pair using an iterative method. A sequence of values was constructed for each of the 6 variables of the base model and 3 active terms (see Supplemental Methods for details), and the model was run for all combinations of each variable and value. For each metabolite/gene pair, two linear correlations (Pearson’s *r*) were calculated between modeled (*P*) and experimental diatom endometabolite data, as well as between modeled bacterial metabolite uptake (*U*) and bacterial transcript expression. Parameter sets that produced significant correlations (*p* < 0.05; *n* = 9) between modeled and experimental data for both endometabolite and gene were saved. The *r* values for each endometabolite/gene pair were averaged and used to compare the overall goodness of fit for each set of model parameters (base, base plus optional active mechanisms in all possible combinations). These *r* values were adjusted to account for the number of terms beyond the base model according to the formula:

$$1 - \left[ {\frac{{\left( {\frac{1}{r}} \right)\left( {n - 1} \right)}}{{n - k - 1}}} \right]$$

where *n* = number of samples and *k* = number of terms. For models with few significant solutions, additional parameter space was explored but did not substantially increase fit.

## Results and discussion

*R. pomeroyi* was inoculated into axenic *T. pseudonana* cultures growing under a naturally oscillating light:dark cycle. After a 2-d pre-incubation to allow the bacteria to assimilate labile metabolites that accumulated during the axenic phase, and thus emphasize synchronized production and consumption dynamics during diel cycles, samples were collected every 6 h for the next 48 h at timepoints corresponding to midnight, mid-morning, noon, and mid-afternoon.

### Diatom metabolome composition

**Fig. 1:**
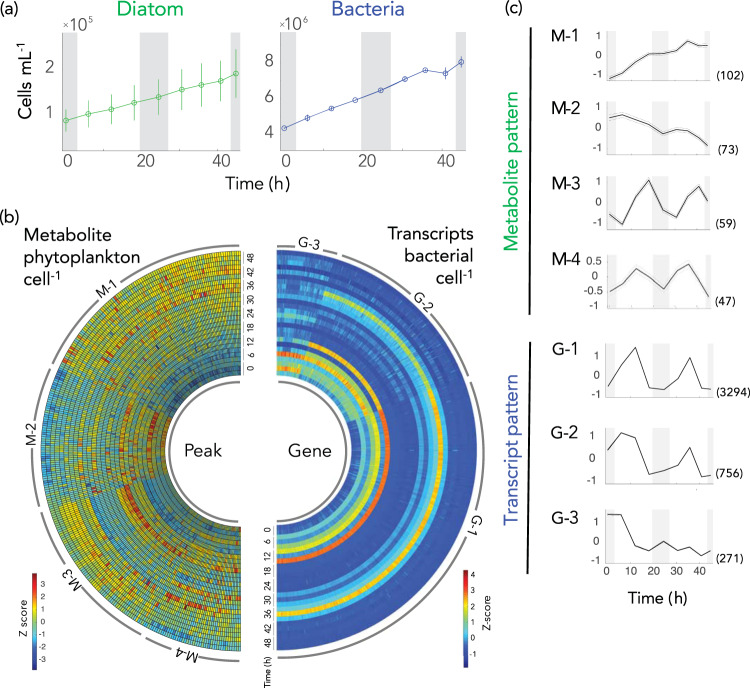
Diel patterns in microbial cell numbers, phytoplankton metabolites, and bacterial transcripts. **a** Cell numbers of co-cultured diatoms and bacteria. **b** Temporal variations in metabolite peak intensity per diatom cell (left) and transcripts per bacterial cell for genes differentially expressed between noon and night (>2 fold-change and adjusted-*p* < 0.05, DESeq2) (right). Values were converted to *Z*-scores and data from each of the three biological replicates are shown. **c** Temporal patterns identified for metabolites (M-1 through M-4) and gene transcription (G-1 through G-3). Dotted lines indicate 95% confidence intervals. The number of metabolite peaks or genes in each cluster is given in parentheses. Grey shading in panels (**a**) and (**c**) indicates night.

^1^H-^13^C two-dimensional NMR characterization of the diatom endometabolome during the 48 h sampling window revealed 281 major peaks. From these, 31 compounds (accounting for 156 peaks) were identified with high confidence (Table 1; see Table S1 and Fig. S1 for detailed annotation and confidence level information). The number of diatom cells increased ~2-fold over the sampling window, from 0.87 to 1.9 × 10^5^ cells mL^−1^ (Fig. 1a); metabolite data were normalized to cell number at each sample time.

**Table 1:** Diatom endometabolites assigned with high confidence in the diel experiment. For detailed information for compound identification and confidence level, see Table S1 and Fig. S1.

Compound category/Sub-category	Compound	Function	Group, temporal pattern
Amine	Trimethylamine N-oxide (TMAO)		n.a.
Amino acid	Alanine	Amino acid metabolism	n.a.
	Arginine	Amino acid metabolism	n.a.
	Asparagine	Amino acid metabolism	M-1 (*p* < 0.001)
	Aspartate	Amino acid metabolism	M-4 (*p* < 0.01)
	Glutamine	Amino acid metabolism	M-4 (*p* < 0.001)
	Glutamate	Amino acid metabolism	n.a.
	Glycine	Amino acid metabolism	M-1 (*p* < 0.01)
	Lysine	Amino acid metabolism	M-1 (*p* < 0.001)
	Proline	Amino acid metabolism/osmoregulation	M-4 (*p* < 0.001)
Amino acid/Branched-chain	Valine	Amino acid metabolism	n.a.
	Isoleucine	Amino acid metabolism	M-1 (*p* < 0.001)
	Leucine	Amino acid metabolism	M-1 (*p* < 0.001)
Amino acid derivative	Glycine betaine	Osmoregulation	M-1 (*p* < 0.05)
	Dimethylglycine		n.a.
	Homarine	Osmoregulation	M-1 (*p* < 0.05)
Amino alcohol	Ethanolamine	Lipid metabolism	M-1 (*p* < 0.01)
Choline	Choline	Lipid metabolism	n.a.
Choline derivative	Phosphorylcholine	Lipid metabolism	M-1 (*p* < 0.001)
Phosphocholine	Glycerophosphocholine	Lipid metabolism	n.a.
Glycerol derivative	Glycerol 3-phosphate	Lipid metabolism	M-1 (*p* < 0.01)
Nucleoside	Adenosine	Nucleic acids/ATP constituent	n.a.
	Guanosine	Nucleic acids/GTP constituent	M-4
	Uridine		M-3 (*p* < 0.001)
Organic acid	3-Hydroxybutyrate	Carbon metabolism	M-2 (*p* < 0.01)
	Acetate	Carbon metabolism	M-2 (*p* < 0.01)
	4-Hydroxyphenylacetate		n.a.
Sugar/*Monosaccharide*	Glucose	Carbon/central energy metabolism	M-3 (p < 0.001)
Sugar/*Polysaccharide*	β(1,3)-glucan	Carbon metabolism/storage	M-3 (*p* < 0.001)
Sulfur compound	Dihydroxypropane-sulfonate (DHPS)	Osmoregulation	M-1 (*p* < 0.001)
	Dimethylsulfonio-propionate (DMSP)	Osmoregulation	M-1 (*p* < 0.001)

Temporal pattern assignments correspond to those in Fig. 1; M-1 = increase, M-2 = decrease, M-3 = diel with a peak at mid-afternoon, and M-4 = diel with a peak at noon. Statistical significance was determined based on linear regression analysis (M-1 and M-2), and RAIN for diel cycles (M-3 and M-4). n.a., not applicable (membership value of >0.5, see text for the detail).

To group metabolite peaks that behaved similarly over the diel cycles, cell-normalized abundance data were clustered by variance-sensitive clustering [44] which identified four patterns (Figs. 1b and 1c; Table 1). Group M-1 consisted of 102 peaks (36%) for which a monotonic increase in intensity dominated the 48 h sampling window (Fig. 1c). The twelve compounds annotated with high confidence from this cluster included amino acids (asparagine, glycine, isoleucine, leucine, and lysine), amino acid derivatives (glycine betaine and homarine), an amino alcohol (ethanolamine), a choline derivative (phosphorylcholine), a glycerol derivative (glycerol-3-phosphate), and the sulfur-containing compounds dihydroxypropanesulfonate (DHPS) and dimethylsulfoniopropionate (DMSP) (Table 1, also see Fig. S2 for individual compounds in each cluster). Metabolite group M-2 was characterized by 73 peaks (26%) for which decrease in intensity over time was the dominant pattern, and included two organic acids (3-hydroxybutyrate and acetate). The two other metabolite clusters exhibited diel concentration patterns that peaked in the light and declined in the dark (Table 1, Fig. S2). Group M-3 contained 59 peaks (21%) that reached their maximum intensities at mid-afternoon (R package RAIN, *p* < 0.001) and included high-confidence annotations of the nucleoside uridine and the carbohydrates glucose and β-1,3-glucan, the latter a subunit of the diatom polysaccharide chrysolaminarin [51]. Group M-4 contained 47 peaks (17%) that reached their maximum intensities at mid-morning or noon (RAIN, *p* < 0.01) and included high confidence annotations for the amino acids aspartate, glutamine, proline, and guanosine. Overall, four distinct temporal patterns in abundance were observed among components of the *T. pseudonana* endometabolome during co-growth with a heterotrophic bacterium under a light regime mimicking that of the surface ocean.

### Bacterial transcription patterns

We next examined concurrent bacterial transcript inventories indicative of metabolite consumption, normalized to cell counts at the time of sampling (Fig. 1a). The total number of transcripts cell^−1^ varied significantly over the diel cycle (One-way ANOVA; *n* = 26, *p* < 0.01) with ~2.5-fold more mRNAs in the mid-morning and noon cells (95 ± 49 and 114 ± 53 mRNAs cell^-1^) relative to mid-afternoon and night (42 ± 11 and 58 ± 25 mRNAs cell^-1^). Correspondingly, the majority of genes had higher transcripts per cell at mid-morning and noon relative to mid-afternoon and night (Fig. S3). This transcript inventory is low compared to exponentially growing *Escherichia coli* (1,350 mRNAs cell^-1^; ref. [52]) but comparable to previous measures for marine bacteria in ocean environments [34].

To identify genes that behaved similarly over time, the per cell transcript inventories for each of the 4,278 protein-encoding genes in the *R. pomeroyi* genome were clustered by variance-sensitive clustering (Fig. 1b, c). Group G-1 consisted of 3,294 genes (76%) exhibiting a diel transcription pattern with a maximum value at noon (Fig. 1c). Group G-2 consisted of 756 genes (17%) exhibiting a diel transcription pattern similar to G-1 but with high values at the first night and mid-morning time points (Fig. 1c). Group G-3 included 271 genes (6%) that did not exhibit a diel pattern, but similar to G-2, the first night and mid-morning values were high (Fig. 1c). We speculate that the higher transcript inventories at initial time points of the G-2 and G-3 clusters reflect incomplete bacterial drawdown of a metabolite accumulating prior to the addition of bacteria.

**Fig. 2:**
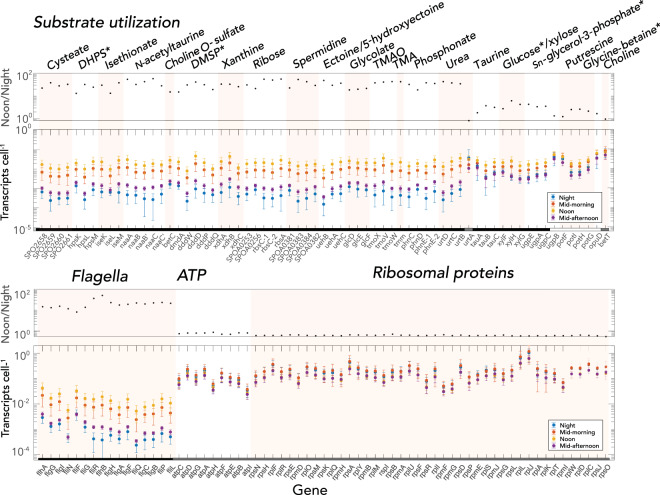
Expression levels of representative *R. pomeroyi* genes encoding transporters or diagnostic catabolic genes (top) and, for comparison, genes encoding flagella, ATPases, and ribosomal proteins (bottom). For each panel, the top plot shows noon to night ratios (black circles), and the bottom plot shows average transcripts cell^-1^ at night, mid-morning, noon, and mid-afternoon. *n* = 8 or 6; error bars indicate standard deviations. Categories of transcription temporal patterns (G-1, black; G-2, white, G-3, grey) are indicated along the x-axis. Asterisks indicate transporters whose target substrate matches an endometabolite identified with high confidence.

Expression levels of *R. pomeroyi* genes annotated with functions in organic carbon influx served as proxies for bacterial substrates made available by phytoplankton extracellular release (372 transporter genes representing 120 transporter systems). In a few cases for which catabolic genes were known but transporter genes were not, we used expression levels of diagnostic catabolic genes as indicators of substrate influx (21 catabolic genes representing 8 catabolic systems) (Table 2). The majority of influx genes exhibited the G-1 diel expression pattern peaking at noon (91%), among them 18 influx systems whose substrates have been experimentally verified [30, 31, 47, 53–55]. Most of these verified G-1 influx systems [15] had large day:night differences in transcript numbers (mean ratio: 33.5 ± 11.7, *n* = 50 for all genes making up the transport systems), with 13- to 58-fold higher per cell transcript inventories at noon relative to night (adjusted *p* < 0.05) (Fig. 2). These systems mediate uptake of osmolytes (ectoine and 5-hydroxyectoine), amines (trimethylamine, trimethylamine-N-oxide, and spermidine), organic sulfur compounds (DHPS, isethionate, cysteate, *N*-acetyltaurine, choline-*O*-sulfate, and DMSP), and glycolate, xanthine, phosphonate, and ribose. The other three experimentally-verified influx systems with G-1 patterns had modest (mean ratio: 3.7 ± 1.2, *n* = 9) but nonetheless significant diel expression differences (adjusted *p* < 0.05) due to high night transcript inventories rather than low noon inventories (Fig. 2); these encode influx of taurine, glucose, and *sn*-glycerol-3-phosphate. Two experimentally verified influx systems in group G-2 encoded uptake of putrescine and glycine betaine, and one in group G-3 encoded choline uptake. The greater expression levels at noon relative to night were also seen in flagella synthesis genes, implying greater bacterial motility at noon, but not for pathways relating to ATP generation and ribosomal protein synthesis, which had constant numbers of transcripts throughout the diel cycle (Fig. 2). Although expression among replicates followed the same patterns though time, variability within a given time point could be substantial. Potential explanations include physiological offsets among replicate diatom cultures and emergence of bacterial subpopulations within replicates [56].

**Table 2:** Bacterial noon/night ratios of transcripts cell^−1^ for genes indicative of metabolite consumption.

	Compound	Gene locus tag	Gene name	Protein function	Noon/Night transcript ratio	Reference
Amide	Urea	SPO1707	*urtD*	ABC transporter, ATP-binding protein	43.0	[25]
		SPO1708	*urtC*	ABC transporter, permease	37.7	
		SPO1709	*urtB*	ABC transporter, permease	34.6	
		SPO1710	*urtA*	ABC transporter, substrate binding	0.8 (n.s.)	
Amine	TMAO	SPO1548	*tmoX*	ABC transporter, periplasmic binding	38.0	[53]
		SPO1550	*tmoV*	ABC transporter, permease protein	41.2	
		SPO1549	*tmoW*	ABC transporter, ATP binding	43.1	
Amine	TMA	SPO1551	*tmm*	TMA monooxygenase	42.6	[25]
Amino acid derivative	Betaine	SPO3186	*opuD*	Glycine-betaine transporter	1.6 (n.s.)	[25]
Amino Acid	**Leucine**	SPO2793	*ivD*	isovaleryl-CoA dehydrogenase	2.6	
		SPO2789	*mccA*	methylcrotonyl-CoA carboxylase, alpha subunit	3.1	
		SPO2790	*mccB*	methylcrotonyl-CoA carboxylase, beta subunit	3.4	
		SPO0390		glutamate/leucine/phenylalanine/valine dehydrogenase	1.2 (n.s.)	
Amino acid derivative	Ectoine/ 5-hydroxyectoine	SPO1146	*uehB*	TRAP transporter, small integral membrane protein	48.4	[31]
		SPO1147	*uehA*	TRAP transporter, large integral membrane protein	29.7	
		SPO1145	*uehC*	TRAP transporter, periplasmic binding	36.7	
Amino Acid	**Proline**	SPO1031		hypothetical protein	21.2	
		SPO2441		ABC transporter, periplasmic betaine/proline-binding	16.4	
		SPOA0231		ABC transporter, periplasmic substrate-binding	43.3	
Choline	Choline	SPO1087	*betT*	Choline transporter	0.9 (n.s.)	[75]
Glycerol derivative	**SN-glycerol-3-phosphate**	SPO0238	*ugpE*	ABC transporter, permease	4.3	[25]
		SPO0239	*ugpA*	ABC transporter, permease	3.3	
		SPO0237	*upgC*	ABC transporter, ATP-binding	3.5	
		SPO0240	*ugpB*	ABC transporter, periplasmic substrate-binding protein	1.3 (n.s.)	
Nuceloside	Xanthine	SPO0654	*xdhA*	Xanthine dehydrogenase, A subunit	33.4	[76]
		SPO0653	*xdhB*	Xanthine dehydrogenase, B subunit	33.0	
		SPO0652	*xdhC*	Xanthine dehydrogenase accessory factor	25.8	
Nucleoside	**Uridine**	SPO2470	*iunH*	inosine-uridine preferring nucleoside hydrolase	9.9	
Organic Acid	**Acetate**	SPO1813	*acs*	acetyl-coenzyme A synthetase	0.9 (n.s.)	
		SPO0325	*phbB*	acetoacetyl-CoA reductase	1.9	
		SPO0326	*phbA*	acetyl-CoA acetyltransferase	1.1 (n.s.)	
Organic acid	Glycolate	SPO3478	*glcD*	glycolate oxidase, GlcD subunit	18.6	[25]
		SPO3479	*glcE*	glycolate oxidase, GlcE subunit	19.9	
		SPO3480	*glcF*	glycolate oxidase, iron-sulfur subunit	21.6	
Phosphonate	Phosphonate	SPO0780	*phnC*	ABC transporter, ATP-binding	32.4	
		SPO0781	*phnD*	ABC transporter, periplasmic phosphonate-binding	18.6	
		SPO0782	*phnE-1*	ABC transporter, permease	38.3	
		SPO0783	*phnE-2*	ABC transporter, permease	35.6	
Polyamine	Putrescine	SPO3469	*potF*	ABC transporter, periplasmic putrescine-binding	1.2	[55]
		SPO3466	*potI*	ABC transporter, permease	2.5	
		SPO3467	*potH*	ABC transporter, permease	2.6	
		SPO3468	*potG*	ABC transporter, ATP-binding	2.1	
Polyamine	Spermidine	SPOA0381		ABC transporter, periplasmic substrate-binding protein	22.7	[55]
		SPOA0383		ABC transporter, permease protein	53.4	
		SPOA0384		ABC transporter, permease protein	42.7	
		SPOA0382		ABC transporter, ATP-binding protein	29.2	
Sugar	Ribose	SPOA0253		ABC transporter, periplasmic substrate-binding	30.9	[47]
		SPOA0254	*rbsC-1*	ABC transporter, permease	54.9	
		SPOA0256		ABC transporter, periplasmic substrate-binding protein	21.4	
		SPOA0257	*rbsC-2*	ABC transporter, permease	50.4	
		SPOA0258	*rbsA*	ABC transporter, ATP-binding	55.7	
Sugar	**Glucose**	SPO0861	*xylF*	ABC transporter, periplasmic substrate-binding	2.7 (n.s.)	[47]
		SPO0862	*xylH*	ABC transporter, permease	6.2	
		SPO0863	*xylG*	ABC transporter, ATP-binding	4.2	
Sulfur compound	Choline-O-sulfate	SPO1083	*betC*	Choline sulfatase	15.1	[75]
Sulfur compound	Cysteate	SPO2658		ABC transporter, periplasmic substrate-binding	22.4	[41]
		SPO2659		ABC transporter, permease	38.3	
		SPO2660		ABC transporter, permease	28.0	
		SPO2661		ABC transporter, ATP-binding	32.7	
Sulfur compound	**Dihydroxypropane-sulfonate (DHPS)**	SPO0591	*hpsK*	TRAP transporter	13.0	[77]
		SPO0592	*hpsL*	TRAP transporter	32.3	
		SPO0593	*hpsM*	TRAP transporter	24.5	
Sulfur compound	**Dimethylsulfonio-propionate (DMSP)**	SPO1913	*dmdA*	DMSP demethylase	15.0	[41]
		SPO0453	*dddW*	DMSP lyase	30.7	
		SPO1703	*dddD*	DMSP lyase	41.3	
		SPO2299	*dddP*	DMSP lyase	31.4	
		SPO1596	*dddQ*	DMSP lyase	19.2	
Sulfur compound	Isethionate	SPO2358	*iseK*	TRAP transporter, periplasmic	30.0	[41]
		SPO2357	*iseL*	TRAP transporter, small permease	13.2	
		SPO2356	*iseM*	TRAP transporter, DctM	38.2	
Sulfur compound	*N*-acetyltaurine	SPO0660	*naaA*	ABC transporter, periplasmic substrate-binding	54.3	[41]
		SPO0661	*naaB*	ABC transporter, permease	32.3	
		SPO0662	*naaB'*	ABC transporter, permease	42.7	
		SPO0663	*naaC*	ABC transporter, ATP-binding	58.2	
		SPO0664	*naaC'*	ABC transporter, ATP-binding	28.2	
Sulfur compound	Taurine	SPO0674	*tauA*	ABC transporter, periplasmic substrate-binding	1.8 (n.s.)	[41]
		SPO0675	*tauB*	ABC transporter, ATP-binding	3.7	
		SPO0676	*tauC*	ABC transporter, permease	3.1	

Bold font indicates the compounds appearing in both the endometabolite and bacterial gene expression datasets.

n.s., not statistically significant (DeSeq2, adjusted *p* > 0.05).

We noticed that *R. pomeroyi* produced two-fold fewer transcripts for substrate acquisition in the mid-afternoon compared to mid-morning (Fig. 2), despite the fact that illumination was identical. Diel oscillations in the relationship between carbon fixation rate and irradiance have been broadly documented for marine phytoplankton in laboratory and field studies, characterized by pre-noon maxima in photosynthesis rates despite equivalent irradiance levels post-noon [57, 58]. Thus the rapid decrease in expression of most bacterial transporters by mid-afternoon suggests that photosynthetic oscillations may also be manifested in phytoplankton extracellular release.

### Control for direct effects of light

Although no light-sensing proteins have been identified in the *R. pomeroyi* genome, we nonetheless checked whether light could be directly driving changes in gene expression. The bacterium was inoculated into spent medium obtained from axenic *T. pseudonana* cultures, representing a natural pool of dissolved metabolites, and exposed for 4 h to one of three light levels that matched co-culture irradiance at noon, mid-morning/mid-afternoon, and night in order to assess differential gene expression with light exposure as the only variable. Only 61 genes in the bacterial culture (1.4% of the *R. pomeroyi* genome) were significantly enriched by one or both light levels compared to the dark treatment (Fig. S4), indicating that the large shifts observed in transporter system expression (Fig. 2) were not directly triggered by light. Nonetheless, the collection of light-enriched genes was not random. Ten genes function in protection against reactive oxygen species (ROS) (Fig. S5, Table S5) which can be formed when light interacts with oxygen or organic compounds [59–61]. Another 16 function in the uptake and metabolism of phosphate (*pstSCAB*, *phoU*), and phosphonate (*phnDEC*, *phnIGHLJN)* (Fig. S5), and were likely under the control of the similarly enriched *phoB* regulatory protein [62–64]. Phosphorus acquisition transcript enrichment was surprising, since phosphate concentrations remain non-limiting for many weeks in this model culture system (>10 µmol L^−1^) [47] and phosphorus availability was identical in all treatments regardless. Higher uptake of phosphorus during morning hours, when photosynthesis rates are low and competition with phytoplankton for phosphorus uptake may be less intense, has been proposed as a niche partitioning strategy for marine bacterioplankton [14, 65]. Based on the concomitant enrichment of phosphorus acquisition genes and ROS-related genes in the absence of light-sensing proteins, we speculate that phosphorus acquisition might be regulated through the cellular detection of ROS dynamics. ROS have known roles in bacterial signaling through redox-sensing transcriptional regulators [66].

### Transcript enrichment versus upregulation

**Fig. 3:**
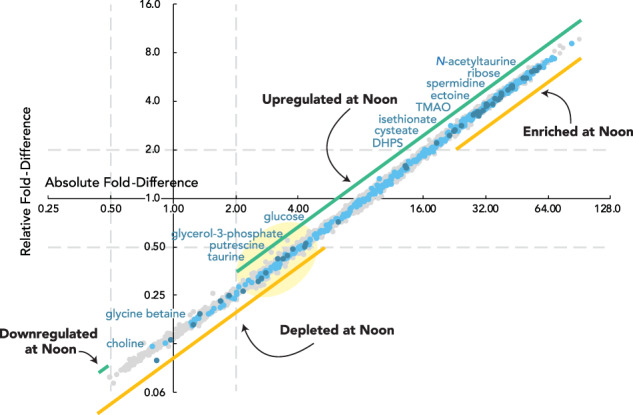
Comparison of fold-difference values for absolute versus relative analysis of noon:night ratios of transcripts. Absolute analysis (x-axis) represents up- or down-regulation of the number of transcripts per bacterial cell. Relative analysis (y-axis) represents enrichment or depletion as a proportion of the transcriptome. Dark blue symbols indicate the transporter genes in Fig. 2; light blue symbols indicate other transporter genes; grey symbols indicate the remaining *R. pomeroyi* genes. Dashed gray lines mark where fold-difference = 2 on each axis (log_2_ units). The light yellow oval highlights genes with per cell transcript inventories that are significantly higher at noon yet account for a significantly lower proportion of the cells’ transcriptome.

Expression of the bacterial influx systems was also calculated as a percent of the transcriptome (Fig. 3), the prevailing analysis approach for RNAseq studies that do not use internal standardization [67]. These calculations identified 57% of influx system genes as having significantly enriched proportions in the noon transcriptome relative to night. In contrast, per cell transcript calculations based on the internal standards identified 94% of influx system genes as having significantly higher transcript inventories at noon relative to night (Fig. 3). These analyses emphasize, on the one hand, the bacterium’s investment in expression of a transporter relative to other cellular functions (percent of transcriptome), and on the other, the absolute number of templates to synthesize the transporter (transcripts per cell). This analysis also revealed that the response of bacterial influx genes is weighted toward higher fold-change compared to the average across all genes for both relative and absolute analyses, implying well-regulated substrate influx. We used per cell bacterial transcript counts and per cell metabolite abundance in the following co-analysis of gene expression and endometabolite data sets.

### Coincidence of diatom metabolite accumulation and bacterial transcription

**Fig. 4:**
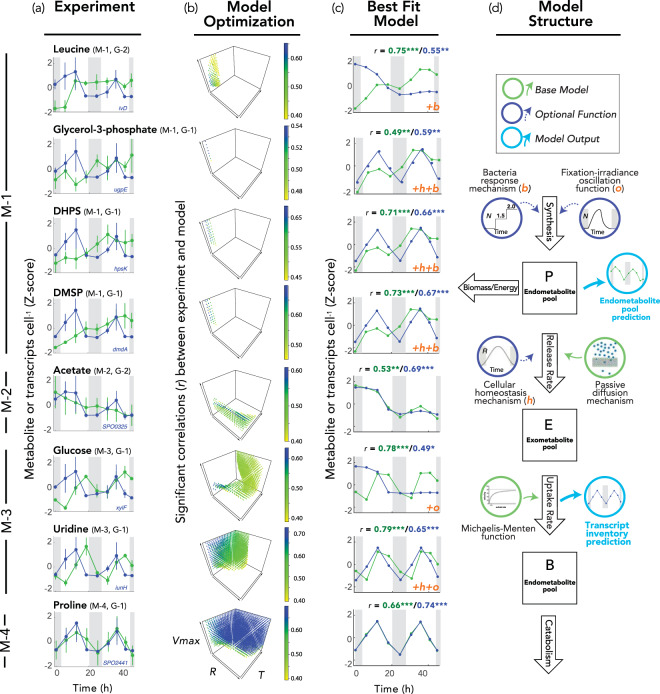
Modeling diatom endometabolite pools and bacterial transcript inventories. **a** Comparison of diel patterns for diatom endometabolite concentration (green symbols) and bacterial transcript inventory for a representative gene encoding uptake or catabolism of the same compound (blue symbols); additional relevant genes are shown in Fig. S6 (mean + standard deviation, *n* = 3 except for the first night where *n* = 2). **b** The base model was optimized with or without optional functions. Each point represents a parameter combination (out of 806,400 tested) for which the model output is significantly correlated with both metabolite and transcript experimental data, and points are colored according to the Pearson’s *r* values for correlations between experimental data and model simulations, averaged for transcript and metabolite datasets. **c** The best fit model based on average adjusted *r*, with individual *r* values given above the plots for metabolite (green font) and transcript (blue font) inventories. Functions added to the base model to achieve the best fit include two physiological balance mechanisms indicated as +*o* (irradiance-fixation oscillation) and +*h* (cellular homeostasis), and a bacterial recognition response indicated as +*b*. *p < 0.05; **p < 0.01; ***p < 0.001. **d** Simulation model structure.

Eight of the bacterial influx systems had target compounds that were also identified with high confidence in the diatom endometabolome, allowing us to compare diel patterns for these matched pairs (Table 2). In all cases, bacterial gene expression followed a diel pattern with peak expression at noon (G-1 or G-2 clusters) (Fig. 4a). The metabolite patterns were more diverse, however. Four metabolites exhibited a continually increasing endometabolome concentration (cluster M-1; leucine, glycerol-3-phosphate, DHPS, and DMSP) (Fig. 4a). One metabolite exhibited a continually decreasing endometabolome concentration (M-2; acetate). Two exhibited diel cycles with mid-afternoon concentration peaks (M-3; glucose and uridine). One exhibited a diel cycle with noon concentration peaks (M-4; proline).

We asked whether the three mechanisms proposed for phytoplankton extracellular release (passive diffusion, physiological balance, and interaction response) could explain the paired patterns in endometabolites (the presumed source) and bacterial transcription (the proxy for extracellular release). A simulation model was established with passive diffusion as the default release mechanism by phytoplankton, governed solely by a metabolite concentration gradient between the inside and outside of cells. Beyond the base model, additional parameters permitted two active physiological balance release mechanisms, fixation-irradiance oscillation and cell homeostasis (for example due to photorespiration or redox imbalance), and one active bacterial interaction mechanism (for bacterial influence over metabolite release rate) (Fig. 4d), and these were tested for improved representation of the experimental data over that of the base model. The model assumed that *R. pomeroyi* transporter systems were regulated by the availability of their substrate, which has been supported in previous studies [30, 31, 53, 55, 68], and that substrates are taken up by the bacteria according to Michaelis-Menten kinetics.

Models in which passive diffusion was the sole mechanism of extracellular release best reproduced the observed concentration pattern for acetate, which steadily decreased through time in the diatom endometabolome (M-2 pattern); and for proline, which had a diel pattern with a peak at noon (M-4 pattern). Adding additional parameters to the base model did not significantly increase fit for these compounds (adjusted *r*, *p* > 0.5; Table S6). Glucose and uridine, which had diel peaks in concentration in the afternoon (M-3 pattern), were best represented in models that included one or both physiological balance mechanisms (fixation-irradiance oscillation and cell homeostasis). Finally, leucine, glycerol-3-phosphate, DMSP, and DHPS, the compounds exhibiting monotonically increasing endometabolite concentrations (M-1 pattern), were best reproduced by the model when the bacterial interaction mechanism was invoked, and may represent shifts to higher steady state concentrations. In contrast to metabolite concentrations, the experimental gene expression data for the 8 compounds all showed a diel peak at noon. While most best-fit models reproduced this transcription pattern, they failed to replicate the noon peaks in expression for acetate, glucose, and leucine (Fig. 4b, c; Fig. S6; Table S6).

The inability of diffusion alone to reproduce the experimental data for the majority of metabolites suggests that passive movement of molecules through cell membranes is insufficient to explain metabolite release [69]. Physiological balance mechanisms represented by the photosynthetic oscillations and cell homeostasis parameters are consistent with evidence that light intensity affects photorespiration rates [3, 70] as well as the biosynthetic balance between metabolite classes (e.g., amino acids versus storage carbohydrates; ref. [71]).

The role of bacterial recognition is more controversial, since the underlying processes by which recognition could be achieved are not yet resolved, nor is it clear how widespread or important such interactions may be in natural microbial communities. Chemicals released from bacteria or bacterial alteration of environmental conditions, such as nutrient pools or ROS, might serve as signals [22]. In previous research, cultured *T. pseudonana* exhibited transcriptional changes consistent with a recognition response acting via leucine-rich repeat (LRR) proteins following addition of *R. pomeroyi* [22]. Similarly, marine diatom *Pseudo-nitzchia multiseries* released tryptophan in response to bacterium *Sulfitobacter* sp. S11 [21] and the bacterium converted tryptophan to the plant hormone indole-3-acetic acid. The model prediction of a role for *R. pomeroyi* in triggering concentration increases of specific *T. pseudonana* endometabolites was tested experimentally by comparing co-cultures to axenic cultures. Three of the four M1 compounds (leucine, glycerol-3-phosphate, and DHPS) had significantly higher concentrations in the diatom endometabolome when incubated with bacteria relative to axenic incubations (Fig. S7). DMSP was the exception, with no significant difference in the *T. pseudonana* endometabolome in the presence or absence of bacteria. DMSP carries out a number of functions in phytoplankton cells, including roles as an osmolyte [72], predator deterrent [73], and ROS scavenger [74], and therefore its regulation in the diatom endometabolome may be more complex than what the model structure could represent (Table S6). Considering these eight focal compounds, computational matching of endometabolite and transcript abundance patterns revealed dynamics consistent with active diatom exudation of labile compounds.

## Conclusions

The quantitative importance of marine phytoplankton-bacteria carbon flux has motivated inquiries into the factors that regulate phytoplankton extracellular release, such as light, temperature, and nutrient limitation [71]. Here we examined diatom internal metabolite accumulation patterns to ask how well they predict external release patterns. We found that blueprints for endometabolite accumulation are diverse (at least four daily patterns), that diatom release strategies vary by molecule, and that active mechanisms involving both diatom physiological balance and bacterial interactions may play key roles. The composition of metabolite release by phytoplankton determines rates and efficiencies of carbon processing by surface ocean bacteria, while the magnitude of metabolite release determines the allocation of recent photosynthate into dissolved versus particulate organic carbon reservoirs, with the former having a lower likelihood of participating in ocean sequestration.

## Supplementary information

Supplemental Material

Table S1

Table S4

Table S5

## Data Availability

Data that support the findings of this study have been deposited in NCBI SRA with BioProject accession number PRJNA649292 (sequencing data), and Metabolomics Workbench with Project ID PR001019, dx.doi.org/10.21228/M80408 (metabolome data).
