# Supplementary material for: Diel investments in metabolite production and consumption in a model microbial system
Source: ISME J. 2021 Dec 17;16(5):1306–17. doi: 10.1038/s41396-021-01172-w (PMC9038784; doi:10.1038/s41396-021-01172-w)
Supplement: Supplementary file 1 — Supplemental Material [file 41396_2021_1172_MOESM1_ESM.pdf]

Supplemental Materials

Diel Investments in Metabolite Production and Consumption in a Model Microbial System

Mario Uchimiya, William Schroer, Malin Olofsson,

Arthur S. Edison, Mary Ann Moran

Supplemental Modeling Methods

Supplemental NMR Methods

Figure S1

Figure S2

Figure S3

Figure S4

Figure S5

Figure S6

Figure S7

Table S2

Table S3

Table S6

Supplemental Modeling Methods

The model consists of three metabolite pools, the phytoplankton endometabolome (*P*), the medium exometabolome (*E*), and the bacterial endometabolome (*B*). The time evolution of these pools was calculated using the following differential equations.

$$\delta_t P = N - T - R \qquad \text{Eq. 1}$$

$$\delta_t E = R - U \qquad \text{Eq. 2}$$

$$\delta_t B = U - C \qquad \text{Eq. 3}$$

Where *N* is the metabolite biosynthesis rate, *R* is release rate from the phytoplankton, and *T* is rate at which endometabolites are allocated for biomass and energy generation by phytoplankton cells. *U* represents bacterial uptake rate of the exometabolome and *C* represents catabolism rate of the metabolite within the bacterial metabolome. These differential equations were solved at times steps of 0.1 h for 10 d using the variable definitions provided below. To simulate the experimental conditions the bacterial term was set to zero until inoculation on day 6.

In the base model *N* is represented by the following cosine equation, gated such that negative values were counted as zeros.

$$N = A * \cos\left(\frac{(t + 0.5) * \pi}{120}\right) + \frac{1}{3} \qquad \text{Eq. 4}$$

where *A* represents the amplitude of *N*, *t* represents the independent variable time in tenths of an hour. *T* was calculated as

$$T = P_0 \, T_F \qquad \text{Eq. 5}$$

where P_0 is the endometabolite concentration at the previous time point and T_F is the fraction of the endometabolite consumed by internal cellular processes during each time step.

R was established as passive diffusion out of the cell

$$R = R_F(P_0 - E_0 G) \qquad \text{Eq. 6}$$

where R_F is the fraction of the endometabolite pool that can be released from the cell in each time step and is multiplied by a term representing the diffusion gradient between endometabolome (P_0) and the exometabolome (E_0) at the previous time step. *G* accounts for the difference in relative volume between the endometabolome and the exometabolome, relating the pool magnitude to a relative concentration.

U is assumed to follow Michaelis-Menten kinetics as

$$U = \frac{V_{max} \; E_0}{k_m \; + \; E_0} \qquad \text{Eq. 7}$$

where V_{max} is the maximum uptake velocity and k_m is the half saturation constant. Finally, C is represented as

$$C = B_0\, C_F \qquad \text{Eq. 8}$$

where B_0 is the magnitude of the bacterial endometabolome at the previous time step and C_F is the fraction of the bacterial endometabolite that is consumed in cellular processes each time step.

In addition to the base model three 'active' terms were added to the model in order to better explain the experimental observations. These active terms include the fixation-irradiation oscillation function (*o*), a cellular homeostasis mechanism (*h*), and the bacterial recognition response term (*b*). The term *o* accounts for asymmetric carbon fixation as light intensity approaches and falls from its peak (48). When *o* is enabled the equation to calculate *N* is modified to

$$N_o = \begin{Bmatrix} -A\sin\left((t+0.5)*\frac{\pi}{120}+\frac{\pi}{6}\right)+\frac{1}{3}, & 0 < t \leq 160 \\ -0.5\,\mathrm{atan}(a_1 * t - a_1 * b_1) + 0.6, & 160 < t \leq (160+H) \\ -0.5\,\mathrm{atan}(a_2 * t - a_2 * b_2) + 0.6, & (160+H) < t \leq 24 \end{Bmatrix} \qquad \text{Eq. 9}$$

Where *A* represents the amplitude of *N*, *t* represents the independent variable time in tenths of an hour, and *H* influences the intensity of this oscillation as the length of time, in tenths of an hour, it takes for *N* to fall to 30% efficiency. To align with the experiment's light-dark cycle the modeled cycle is: dark from 0:00 hours to 08:00 hours, and light from 08:00 hours to 24:00 hours, with peak light intensity at 16:00 hours. The coefficients *a* and *b* were solved for as

$$a = \frac{\tan(-2N+1.2) - \tan(-2N_0+1.2)}{t - t_0} \qquad \text{Eq. 10}$$

$$b = \frac{-\tan(-2N+1.2)}{a} + t_0 \qquad \text{Eq. 11}$$

so that the function *N* is continuous, where N_0 is the initial value of *N* for each time range and t_0 is the initial time.

The term *h* increases release during periods of high light intensity. When *h* is active the term *R* in Eq. 1 and 2 is multiplied by *p* calculated as

$$h = \cos\left(\frac{(t+0.5)*\pi}{120} + \frac{80*\pi}{120}\right) + 1 \qquad \text{Eq. 12}$$

Finally, the bacterial recognition response term (*b*) increases the production in response to the presence of bacteria. When *b* is active, it represents a constant factor multiplied by *N* in Eq. 1 when bacteria are present (after day 6).

The model was fit to each metabolite/gene pair by an iterative method. Value ranges were selected for each parameter based on preliminary testing of the model. The model was run with all possible combinations of these parameters (806,400 total parameter combinations). For each parameter combination, model output of *P* and *B* was tested for correlation with experimental endometabolite and transcript data, respectively. If both metabolite and gene correlations were positive and significant, the mean of both correlation coefficients (*r*) was calculated. The parameter set that yielded the highest mean *r* value was used as the best fit model for each metabolite.

Parameter values used in iterative model fitting are given below. Type indicates whether each parameter is part of the base model or an optional active term. *n* indicates the total number of values for each parameter

Parameter	Type	Values	*n*
R_F	Base	0.001, 0.002, … , 0.02	20
T_F	Base	0.001, 0.002, … , 0.02	20
V_{max}	Base	0.3, 0.41, … , 1.5	14
K_m	Base	2, 4, 6	3
G	Base	0, 0.05, 0.15	3
A	Base	0.1, 0.667	2
o	Active	‘No', 'Yes'	2
h	Active	‘No', 'Yes'	2
b	Active	‘No', 'Yes (factor of 2)'	2

Supplemental NMR Methods

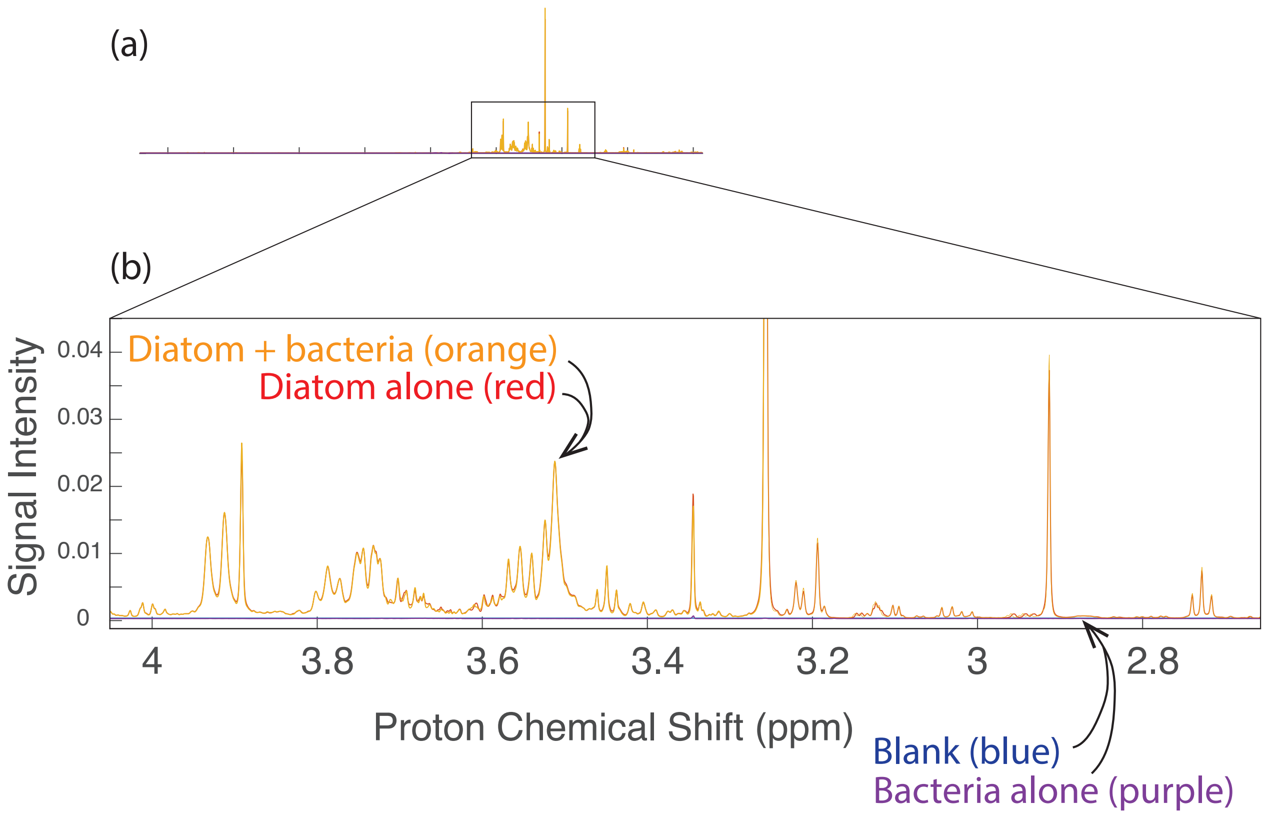

Analysis of the contribution of bacterial metabolites to the 2.0-μm fraction.

In the co-culture treatments (diatom plus bacteria), we collected diatom endometabolites using 2.0–μm filters. Here we describe an experimental check for potential contamination by metabolites originating from bacteria that were trapped on the filters.

Three sample types (diatom alone, bacteria alone, and diatom plus bacteria; n=3) were filtered onto 2.0-μm filters. Diatom and bacterial strains, cell number (10^5 and 10^6 cells ml^{-1} for diatom and bacteria, respectively), media, filters, and the extraction protocol used were identical to those in the original experiment. Analysis of metabolites from cells caught on the filters using a 1-dimensional proton NMR experiment (panel a) shows little difference between bacteria-alone treatment and the blank (panel b, blue and purple lines at the baseline), indicating bacterial metabolites captured by 2.0-μm filters are minimal. In addition, the spectra of the diatom+bacteria treatment overlapped with diatom-alone (panel b, orange and red lines), further indicating that metabolites extracted from the 2.0–μm filters are dominated by those originating from the diatom endometabolome with a negligible contribution by trapped bacterial cells .

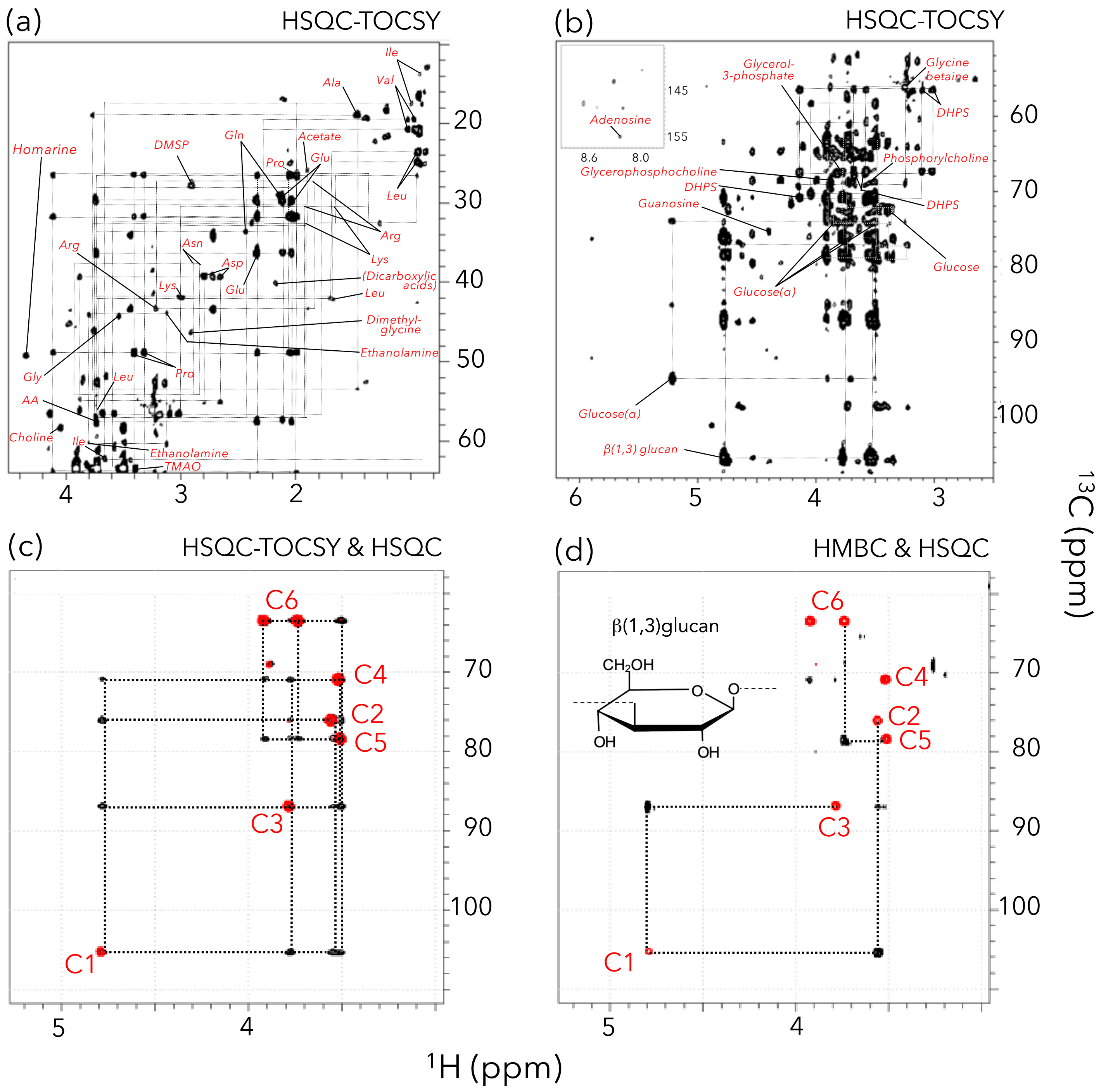

Figure S1. Diatom endometabolome annotation methods. Representative peak(s) for compounds indicated on HSQC-TOCSY spectra (a and b). Additional structural validation (e.g., for polysaccharide β-1,3-glucan) by HSQC-TOCSY (c) and HMBC experiments (d). Peaks from HSQC experiments are overlaid and colored in red. A complete compound list is provided in Table 1, and chemical shift information used for annotation is provided in Table S1. 3-Hydroxybutyrate, 4-hydroxyphenylacetate, and uridine are not visible in a and b due to relatively low intensities. AA, amino acid alpha carbon.

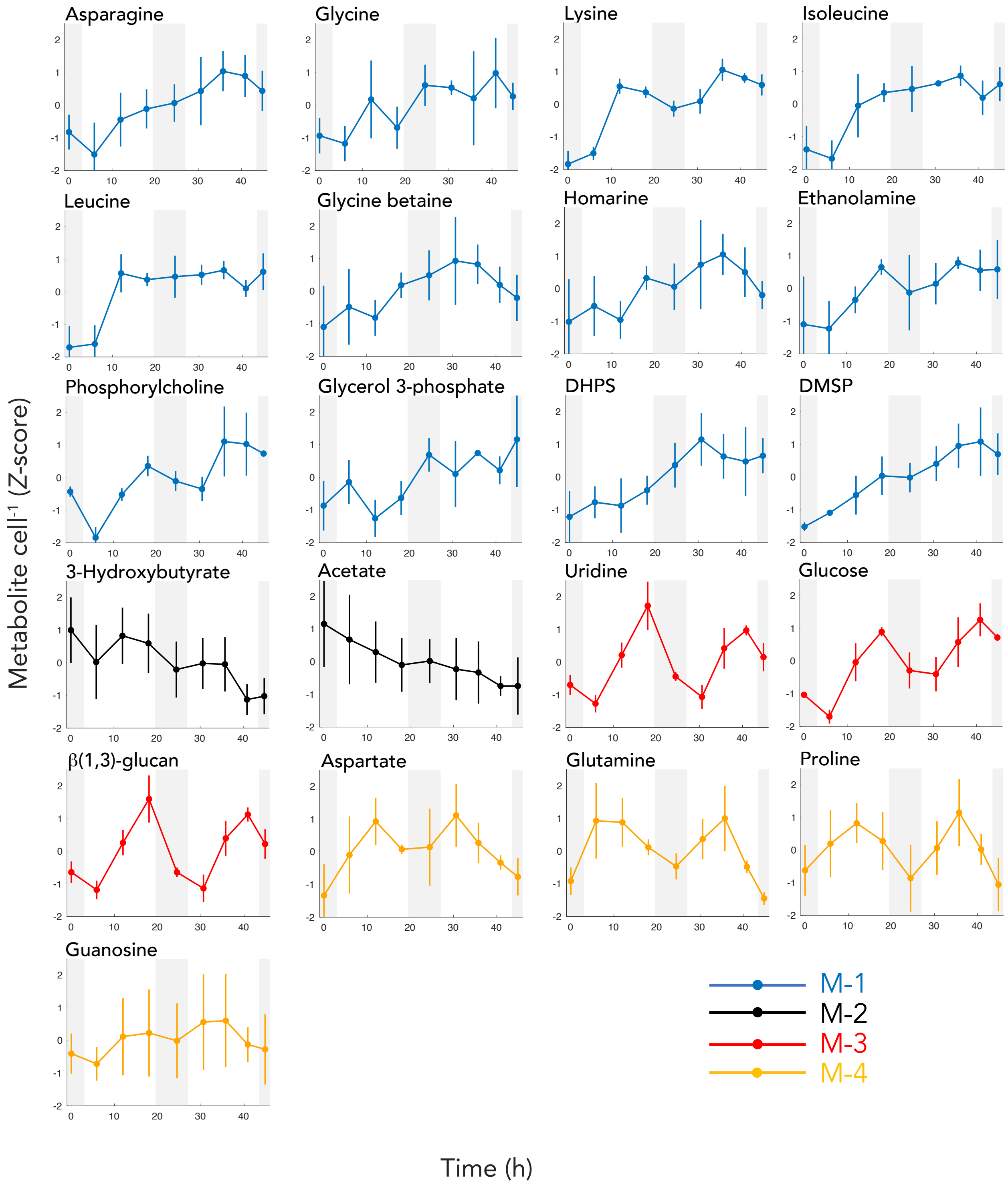

Figure S2. Temporal patterns in 21 diatom endometabolites annotated with high confidence and having significant membership values in a temporal cluster (M-1 through M-4). Metabolite abundance cell^{-1} is shown as *Z*-scores for one representative non-overlapping NMR peak (see also Table S1). Error bars indicate standard deviation; *n* = 3.

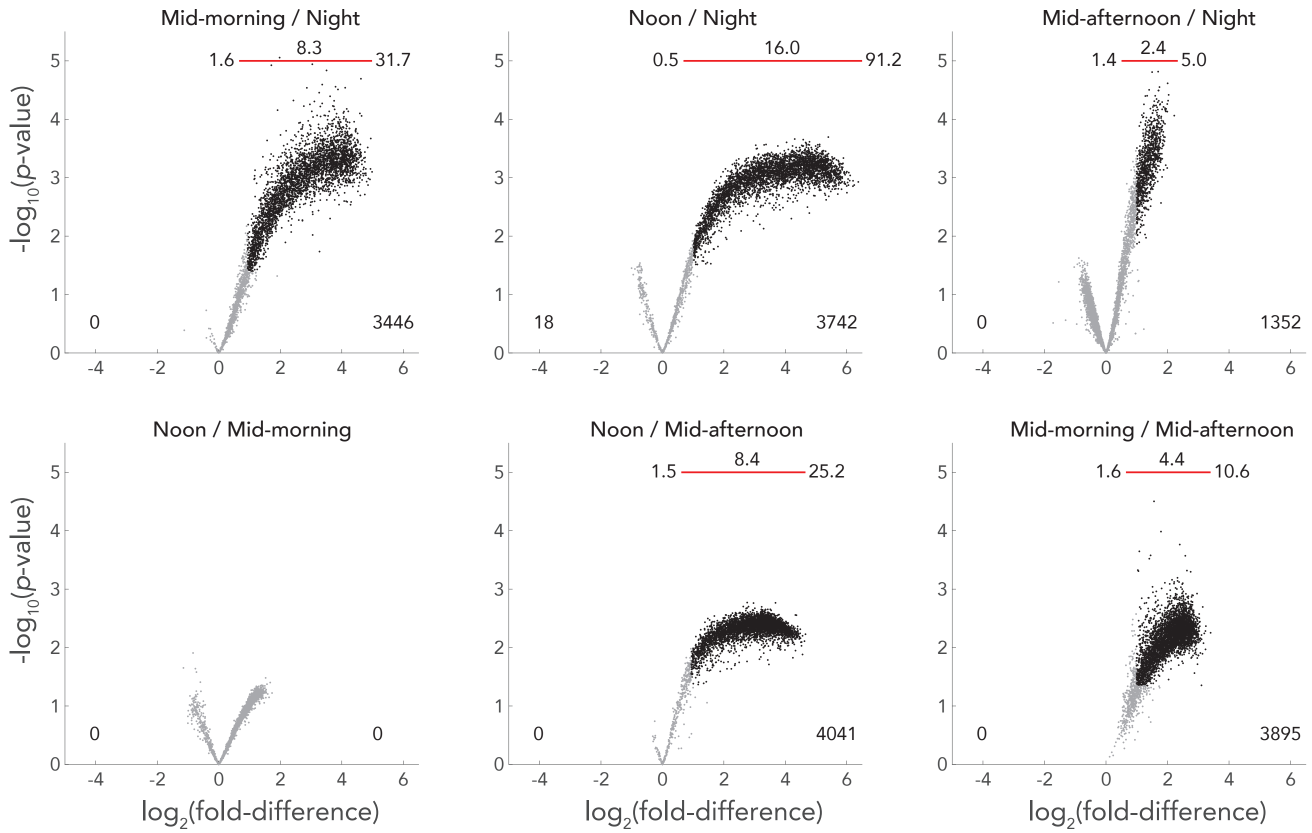

Figure S3. Pair-wise comparison between sample times of bacterial gene expression as transcripts cell^{-1} in the co-culture experiment. Average, minimum-, and maximum difference values for differentially expressed genes (black symbols) are shown above the plots. Numbers on the bottom left and right indicate the number of differentially expressed genes (adjusted-$p \leq 0.05$ and fold-difference ≥ 2).

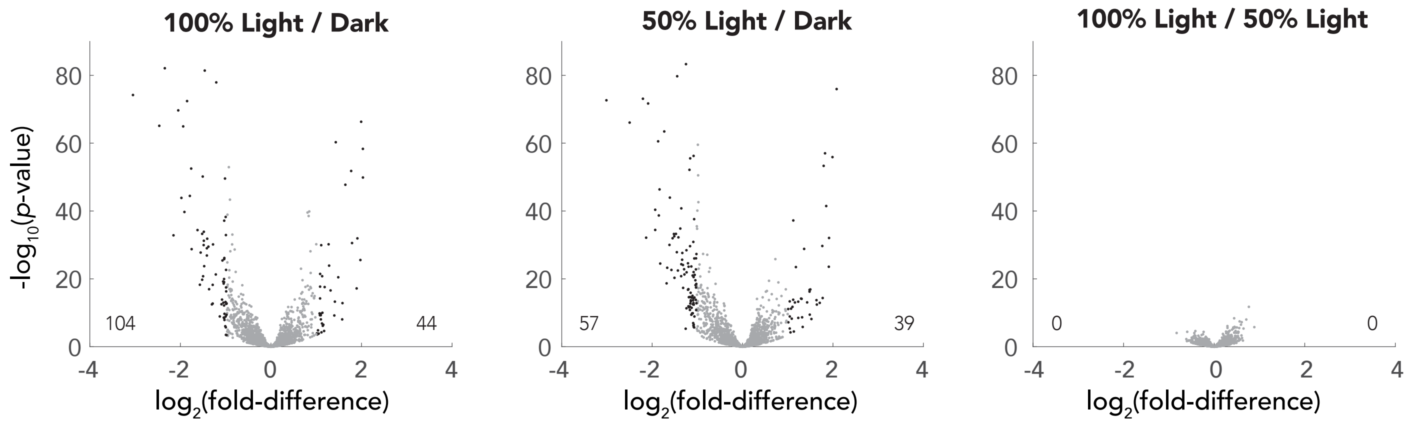

Figure S4. Effects of light exposure on bacterial gene expression as percent of transcriptome. Three light levels corresponding to those at the sample times in the co-culture experiment were examined: 100%, light level at noon; 50%, light level at mid-morning and mid-afternoon; dark, light level at night. Differentially expressed genes with fold-difference of ≥ 2 and DESeq2 adjusted-$p \leq 0.05$ are shown as black symbols.

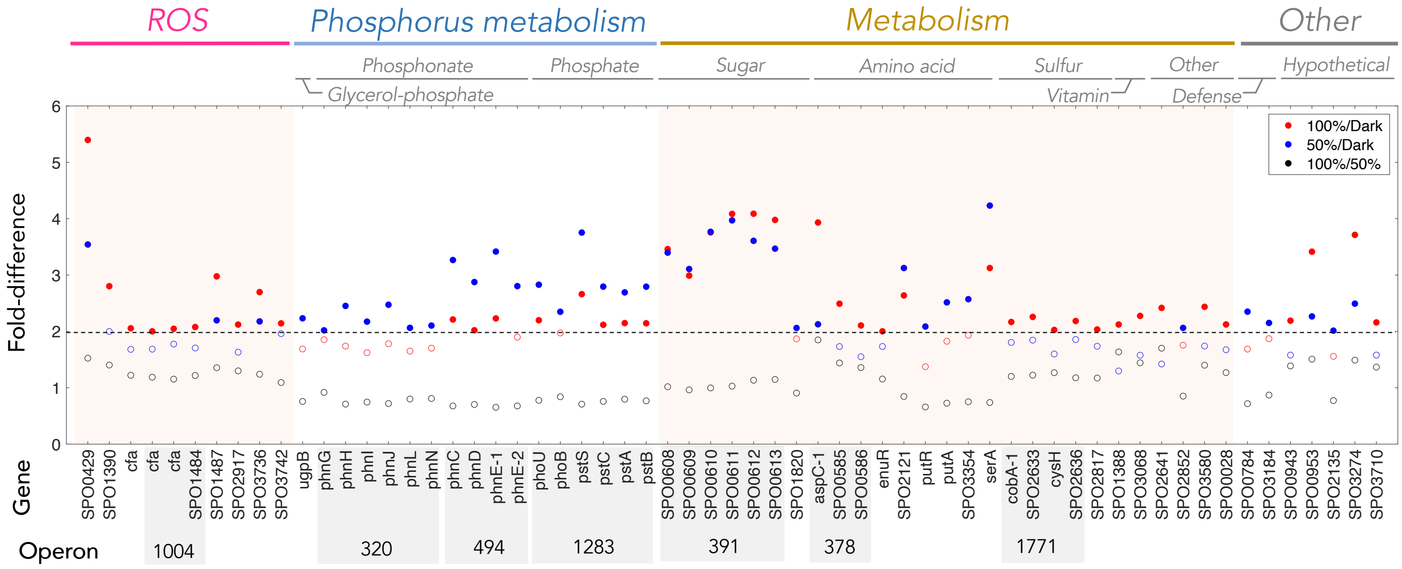

Figure S5. Direct effects of light on gene expression by *R. pomeroyi*. Filled circles indicate differentially expressed genes (fold-difference ≥2 and DESeq2 adjusted-*p* ≤ 0.05) at 100% light level relative to dark (red symbols), 50% light level relative to dark (blue symbols), and 100% light level relative to 50% light level (black symbols). Open circles with the same color codes represent comparisons that were not significantly different. All 61 genes enriched in the presence of light are included, and detailed gene information is given in Table S5. The horizontal line represents 2 fold-difference.

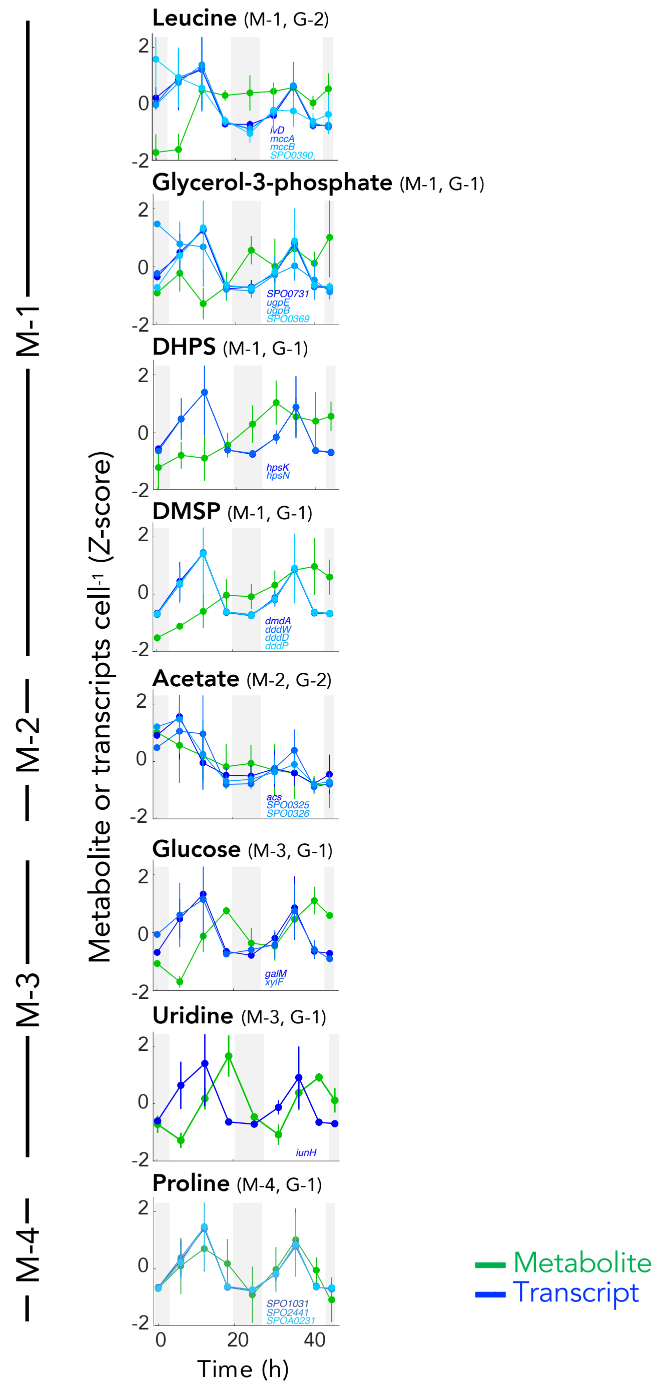

Figure S6. Temporal concentration changes in the eight diatom endometabolites (green) plotted with bacterial transcript inventories (transcript cell^{-1}) for all identified genes encoding uptake or catabolism of the same compound (blue shades). Error bars indicate standard deviation (n = 3, except for genes in the first night where n = 2).

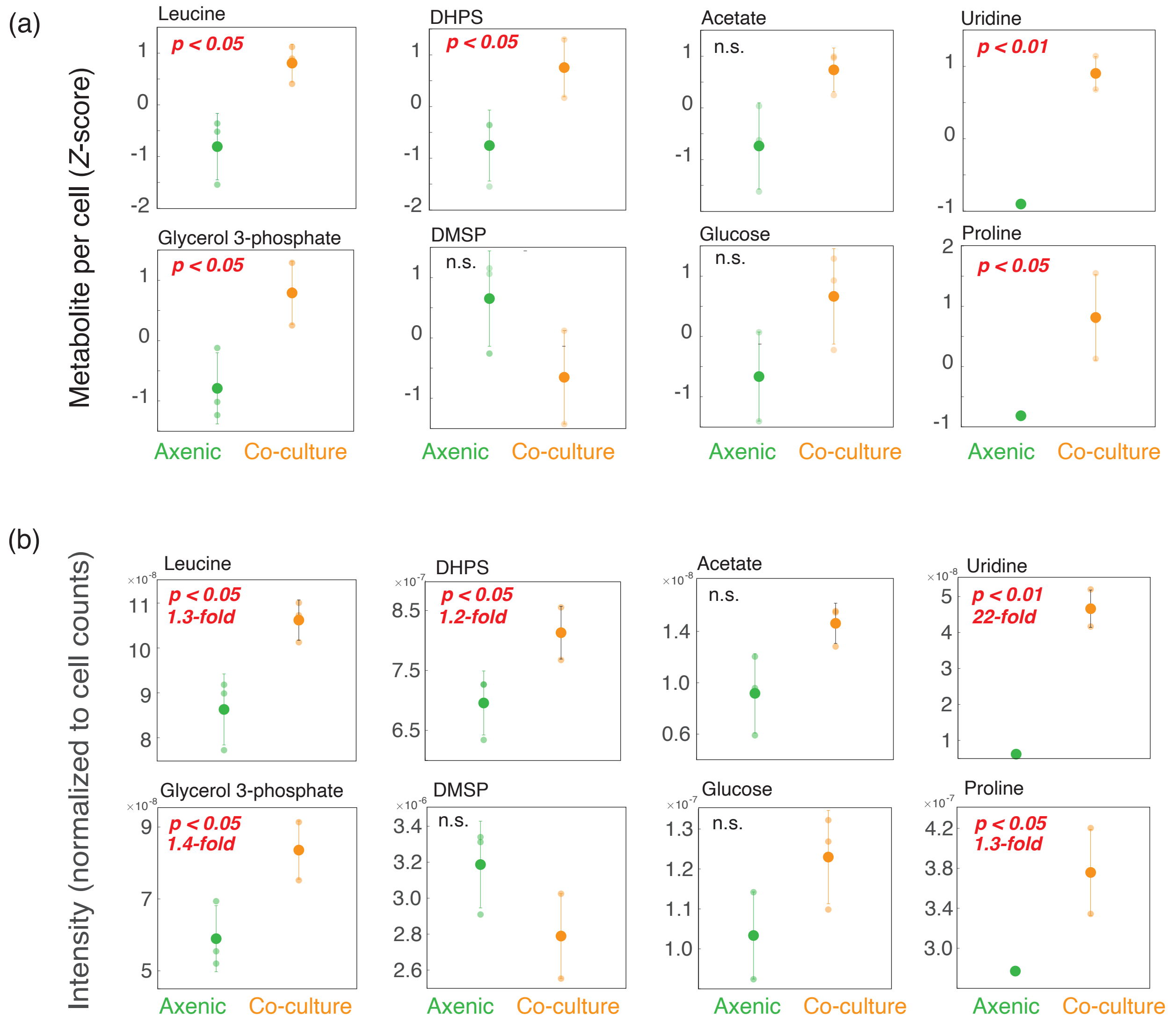

Figure S7. Effects of the presence of bacteria on diatom endometabolite concentration after 48 h. (a) Metabolite per cell values in axenic diatoms (green) and diatoms in co-culture with bacteria (orange) are scaled to *Z*-scores ($n = 3$). *p*-values for differences that are statistically significant are shown in bold red font. (b) Corresponding figures for intensity values, with fold-differences between co-culture and axenic treatments indicated for comparisons that are statistically significant.

Table S2. Instrument settings for NMR experiments.

Experiment (program name by Bruker)	Corresponding figure and table	Spectral width/offset (ppm)		Size of FID		Number of scans
		f2	f1	f2	f1	
^{1}H-^{13}C HSQC (hsqcetgpprsisp2.2)	Figure 1, 4, S2, S6, and S7	14.2/4.7	40.0/68.0	1024	96	16
	Fig. S1; Table S1	14.2/4.7	159.9/75.0	1024	256	256
^{1}H-^{13}C HSQC-TOCSY (hsqcdietgpsisp.2)	Fig. S1	14.2/4.7	189.9/90.0	1024	256	256
^{1}H-^{13}C HMBC (hmbcetgpl2nd)	Fig. S1	12.0/4.7	140/70.0	5768	390	96

Table S3. Summary of transcriptome sequence data. #reads, raw reads. #QC, number of reads passing quality control. QC%, percent of reads passing quality control. rRNA, number of reads identified as rRNA, %rRNA, percent of reads identified as rRNA, MTST5, number of reads identified as internal standard 5. MTST6, number of reads identified as internal standard 6. MTST_total, sum of MTST5 and MTST6 reads. std%, percent of reads identified as internal standards. #ReadsLeft, mRNA reads remaining for analysis. n.a., not applicable.

ID	Sample	#reads	#QC	QC%	rRNA	rRNA%	MTST5	MTST6	MTST_total	std%	#ReadsLeft
E05	Co-culture experiment, Night1-1	13,498,405	12,363,245	91.59	3,406,623	27.6	166,618	608,592	775,210	6.3	8,181,412
F04	Co-culture experiment, Night1-2	15,502,853	14,168,744	91.39	2,786,395	19.7	140,544	482,014	622,558	4.4	10,759,791
F02	Co-culture experiment, Mid-morning1-1	16,837,562	15,353,979	91.19	1,712,502	11.2	129,664	403,709	533,373	3.5	13,108,104
E01	Co-culture experiment, Mid-morning1-2	17,807,607	16,244,554	91.22	3,132,687	19.3	176,739	576,341	753,080	4.6	12,358,787
E10	Co-culture experiment, Mid-morning1-3	19,910,294	18,205,466	91.44	2,953,724	16.2	115,098	270,503	385,601	2.1	14,866,141
F03	Co-culture experiment, Noon1-1	19,275,856	17,722,932	91.94	1,164,010	6.6	115,767	324,058	439,825	2.5	16,119,097
D08	Co-culture experiment, Noon1-2	19,685,509	18,037,235	91.63	1,825,730	10.1	312,628	990,970	1,303,598	7.2	14,907,907
E02	Co-culture experiment, Noon1-3	19,408,658	17,720,533	91.30	966,890	5.5	81,101	241,754	322,855	1.8	16,430,788
E11	Co-culture experiment, Mid-afternoon1-1	20,885,480	19,054,538	91.23	3,581,342	18.8	324,456	959,900	1,284,356	6.7	14,188,840
E12	Co-culture experiment, Mid-afternoon1-2	17,557,260	16,073,027	91.55	3,914,892	24.4	261,021	719,276	980,297	6.1	11,177,838
E03	Co-culture experiment, Mid-afternoon1-3	16,977,882	15,523,460	91.43	1,406,118	9.1	348,476	1,057,642	1,406,118	9.1	12,711,224
D12	Co-culture experiment, Night2-1	23,851,789	22,079,516	92.57	7,073,693	32.0	233,505	1,014,268	1,247,773	5.7	13,758,050
F07	Co-culture experiment, Night2-2	19,469,653	17,836,736	91.61	3,663,942	20.5	197,821	734,821	932,642	5.2	13,240,152
F09	Co-culture experiment, Night2-3	31,934,931	29,269,343	91.65	5,338,425	18.2	479,384	1,609,432	2,088,816	7.1	21,842,102

D10	Co-culture experiment, Mid-morning2-1	20,554,827	18,824,484	91.58	2,744,962	14.6	186,059	571,690	757,749	4.0	15,321,773
D11	Co-culture experiment, Mid-morning2-2	13,279,747	12,174,740	91.68	1,927,329	15.8	203,698	551,681	755,379	6.2	9,492,032
E04	Co-culture experiment, Mid-morning2-3	14,605,987	13,367,467	91.52	2,215,170	16.6	109,203	347,448	456,651	3.4	10,695,646
E06	Co-culture experiment, Noon2-1	19,722,654	18,019,139	91.36	1,623,225	9.0	136,858	395,528	532,386	3.0	15,863,528
E08	Co-culture experiment, Noon2-2	28,885,375	26,482,283	91.68	1,079,553	4.1	117,326	323,826	441,152	1.7	24,961,578
F06	Co-culture experiment, Noon2-3	21,993,618	20,024,426	91.05	2,301,998	11.5	165,550	518,511	684,061	3.4	17,038,367
E09	Co-culture experiment, Mid-afternoon2-1	22,556,906	20,538,846	91.05	3,499,739	17.0	334,172	1,166,834	1,501,006	7.3	15,538,101
F08	Co-culture experiment, Mid-afternoon2-2	19,472,311	17,798,623	91.40	2,893,773	16.3	191,917	749,108	941,025	5.3	13,963,825
D09	Co-culture experiment, Mid-afternoon2-3	13,658,878	12,514,100	91.62	3,167,132	25.3	266,044	762,316	1,028,360	8.2	8,318,608
E07	Co-culture experiment, Night3-1	18,841,406	17,419,067	92.45	6,766,726	38.8	147,411	492,285	639,696	3.7	10,012,645
F01	Co-culture experiment, Night3-2	15,647,403	14,312,249	91.47	2,959,371	20.7	145,088	638,394	783,482	5.5	10,569,396
F05	Co-culture experiment, Night3-3	16,962,656	15,604,901	92.00	4,147,196	26.6	220,930	803,400	1,024,330	6.6	10,433,375
0-1	Direct light experiment, light level 0%-1	25,586,505	24,300,490	94.97	6,613,045	27.2	n.a.	n.a.	n.a.	n.a.	17,687,445
0-2	Direct light experiment, light level 0%-2	31,663,480	30,051,703	94.91	10,646,884	35.4	n.a.	n.a.	n.a.	n.a.	19,404,819
0-3	Direct light experiment, light level 0%-3	26,282,421	24,902,417	94.75	9,821,337	39.4	n.a.	n.a.	n.a.	n.a.	15,081,080
50-1	Direct light experiment, light level 50%-1	32,174,743	30,533,445	94.90	6,613,045	21.7	n.a.	n.a.	n.a.	n.a.	21,524,552
50-2	Direct light experiment, light level 50%-2	29,926,762	28,405,710	94.92	10,646,884	37.5	n.a.	n.a.	n.a.	n.a.	17,997,524
50-3	Direct light experiment, light level 50%-3	23,998,399	22,748,705	94.79	9,821,337	43.2	n.a.	n.a.	n.a.	n.a.	11,800,777

100-1	Direct light experiment, light level 100%-1	26,526,300	25,152,623	94.82	6,613,045	26.3	n.a.	n.a.	n.a.	n.a.	15,731,031
100-2	Direct light experiment, light level 100%-2	30,378,955	28,845,621	94.95	10,646,884	36.9	n.a.	n.a.	n.a.	n.a.	16,872,089
100-3	Direct light experiment, light level 100%-3	33,568,541	30,855,170	91.92	9,821,337	31.8	n.a.	n.a.	n.a.	n.a.	19,179,745

Table S6. Summary of the model output. Eight parameter combinations were modeled and the results evaluated using Pearson's correlation between the model and experiment values. A mean Pearson's *r* for a metabolite and corresponding per cell transcripts of the bacterial uptake gene was calculated, and the highest *r* over a range of parameters is shown. The best fit model for each compound is indicated in bold, after adjustment for parameter number. For details of model structure and parameters, see Methods and Supplementary Methods. n.s., no significant output (p>0.05). *o*, carbon fixation-irradiance oscillation function; *h*, cellular homeostasis mechanism; *b*, bacterial response mechanism.

Model type	Acetate	DHPS	Glycerol 3-phosphate	DMSP	Proline	Glucose	Uridine	Leucine
Base model	**0.609**	n.s.	n.s.	0.480	**0.699**	0.475	0.509	0.526
Base model +*o*	0.587	n.s.	n.s.	0.443	0.656	**0.635**	0.613	0.559
Base model +*h*	0.631	n.s.	n.s.	n.s.	0.700	n.s.	n.s.	n.s.
Base model +*b*	0.571	n.s.	n.s.	0.503	0.692	0.599	0.493	**0.652**
Base model +*o* +*h*	0.632	n.s.	n.s.	0.554	0.673	0.638	**0.720**	0.558
Base model +*o* +*b*	0.494	n.s.	n.s.	0.443	0.654	0.665	0.576	0.660
Base model +*h* +*b*	0.555	**0.687**	**0.540**	**0.698**	0.700	0.524	n.s.	0.564
Base model +*o* +*h* +*b*	0.559	0.707	0.587	0.733	0.673	0.690	0.715	0.636
